# Conducting national burden of disease studies and knowledge translation in eight small European states: challenges and opportunities

**DOI:** 10.1186/s12961-022-00923-1

**Published:** 2022-10-21

**Authors:** Sarah Cuschieri, Ala’a Alkerwi, Mary Economou, Jane Idavain, Taavi Lai, Tina Lesnik, Caine Meyers, Hanen Samouda, Inga Dóra Sigfúsdóttir, Natasa Terzic, Lilian Tzivian, Elena Pallari

**Affiliations:** 1 Department of Anatomy, Faculty of Medicine and Surgery, University of Malta, RM 425 Biomedical Building, Msida, Malta; 2 Ministry of Health, Directorate of Health, Service Epidemiology and Statistics, Luxembourg, Luxembourg; 3 Department of Nursing, School of Health Sciences, Cyprus University of Technology, Limassol, Cyprus; 4 National Institute for Health Development, Tallinn, Estonia; 5 Fourth View Consulting, Tallinn, Estonia; 6 National Institute of Public Health, Ljubljana, Slovenia; 7 Icelandic Centre for Social Research and Analysis, Reykjavik, Iceland; 8 Precision Health Department, Luxembourg Institute of Health, Strassen, Luxembourg; 9 Institute of Public Health of Montenegro, Podgorica, Montenegro; 10 Faculty of Medicine, University of Latvia, Riga, Latvia; 11 Health Innovation Network, Minerva House, 5 Montague Cl, London, SE1 9BB United Kingdom

**Keywords:** Burden of disease, Knowledge translation, Small countries, Research

## Abstract

**Background:**

Several countries across Europe are engaging in burden of disease (BoD) studies. This article aims to understand the experiences of eight small European states in relation to their research opportunities and challenges in conducting national BoD studies and in knowledge translation of research outputs to policy-making.

**Methods:**

Countries participating in the study were those outlined by the WHO/Europe Small Countries Initiative and members of the Cooperation in Science and Technology (COST) Action CA18218 European Burden of Disease Network. A set of key questions targeting the research landscape were distributed to these members. WHO’s framework approach for research development capacities was applied to gain a comprehensive understanding of shortages in relation to national BoD studies in order to help strengthen health research capacities in the small states of Europe.

**Results:**

Most small states lack the resources and expertise to conduct BoD studies, but nationally representative data are relatively accessible. Public health officials and researchers tend to have a close-knit relationship with the governing body and policy-makers. The major challenge faced by small states is in knowledge generation and transfer rather than knowledge translation. Nevertheless, some policy-makers fail to make adequate use of knowledge translation.

**Conclusions:**

Small states, if equipped with adequate resources, may have the capacity to conduct national BoD studies. This work can serve as a model for identifying current gaps and opportunities in each of the eight small European countries, as well as a guide for translating country BoD study results into health policy.

**Supplementary Information:**

The online version contains supplementary material available at 10.1186/s12961-022-00923-1.

## Background

Burden of disease (BoD) studies follow a methodological framework that considers the effects of morbidity and premature mortality due to injuries, diseases and risk factors occurring in a country or a region. The metric used is disability-adjusted life-years (DALYs), which combines two indicators: years lived with disability (YLD) and years of life lost (YLL) [1]. BoD studies provide the foundation for policy-makers to plan and prioritize health policies at a population or regional level. These indicators can also be used as an evaluation tool to assess the effectiveness of public health interventions.

The Global Burden of Disease (GBD) study conducted by the Institute for Health Metrics and Evaluation (IHME) provides global BoD estimates based on a comprehensive methodology of specific assumptions and complex statistical models [2]. Nevertheless, there are concerns about the accuracy of this methodology, with recommendations for developing country-specific estimates [3, 4]. To achieve this, national or regional BoD experts and resources (financial, human and infrastructure), including access to good-quality data, are required. Over the past few years, national BoD studies have been conducted across a number of countries in Europe. They can serve as a road map for other counties or regions to replicate their methodology [5–7]. Additionally, methodological guidelines for understanding, conducting and interpreting BoD studies have been developed [8–10]. In October 2019, the European Burden of Disease Network was established within the framework of the Cooperation in Science and Technology (COST) Action CA18218, with the aim of serving as a technical platform for integrating and building capacity for BoD assessment in Europe [11].

The guidelines and studies available to date have mostly targeted large countries rather than small states. A similar scenario is found when it comes to application of the findings (knowledge translation) of BoD by policy-makers and politicians. Although one can argue that small states follow the same principles and characteristics as large countries despite a much smaller population size, this is not the case. Small states share unique challenges and advantages in conducting national studies and knowledge translation [12–14]. Thus far, however, the challenges, capacities and opportunities of small states in conducting BoD studies have rarely been explored.

The aim of this study is to provide a descriptive understanding of the experiences of eight small European states, namely Cyprus, Estonia, Iceland, Latvia, Luxembourg, Malta, Montenegro and Slovenia, in relation to their research opportunities, challenges and capacity in conducting national BoD studies and in knowledge translation of research outputs to policy-making. This analysis will facilitate benchmarking, identification of best practices and lessons to be learned within and across these countries.

## Methods

Eight small states were considered for this study as defined by the WHO/Europe Small Countries Initiative [15]. All authors of this study are representatives of small states and participating members of the COST Action CA18218 European Burden of Disease Network, who provided the data collection. A total of 32 participating COST members are from small countries. The members making up the management committee of this COST action were contacted by the lead researcher (SC) to participate in this study.

**Fig. 1:**
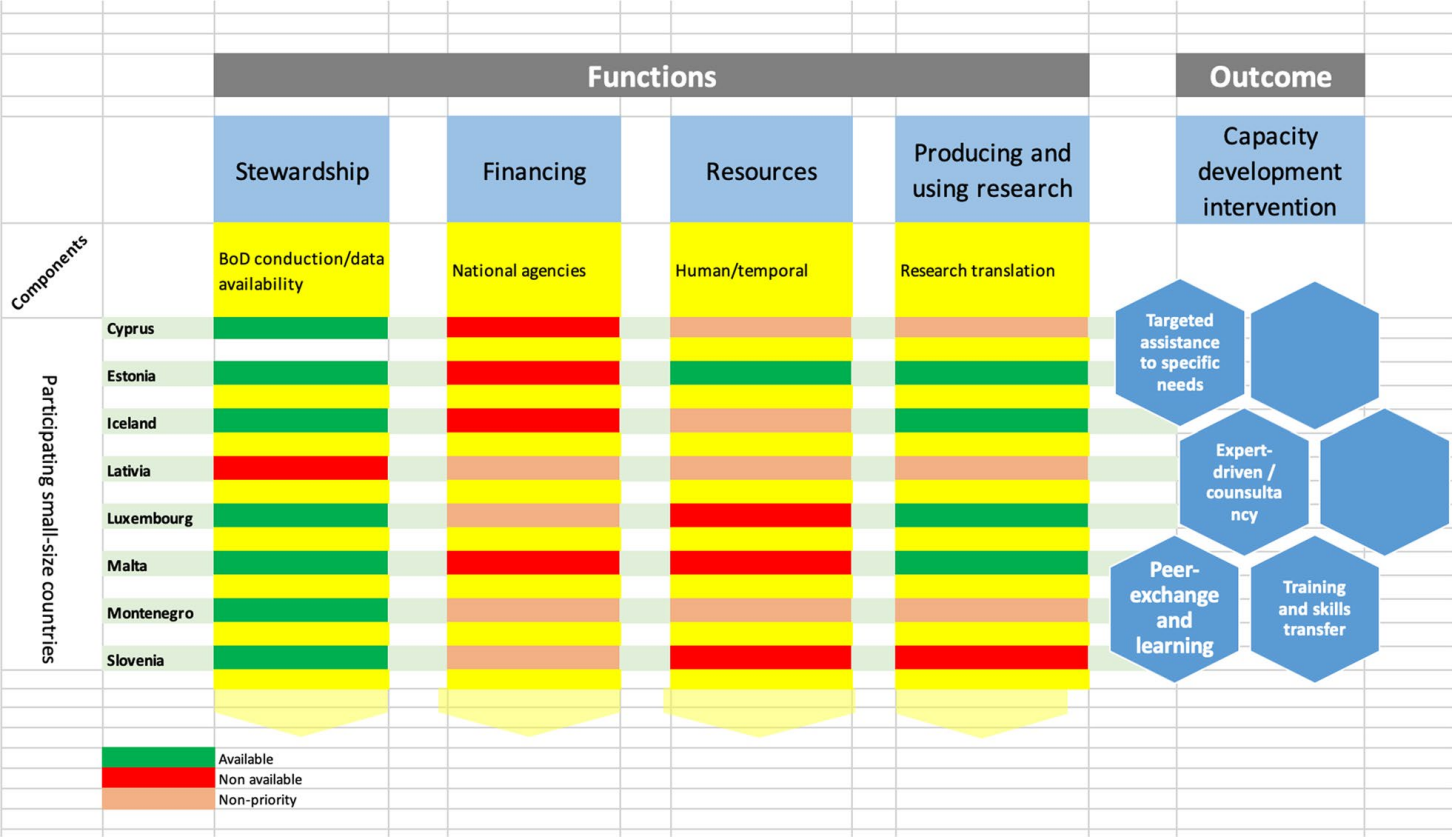
Research landscape in eight small states with respect to functions and outcome based on WHO conceptual framework, 2003

The conceptual framework suggested by WHO in 2003 [16] was used as a foundation on which to base our operational description and analyses of the existing health research systems of the eight contributing countries. This framework is based on four principal functions, namely (i) stewardship, (ii) financing, (iii) building human and physical resources, and (iv) producing and using research, with each composed of several operational components (Fig. 1). For this study, the four functions were defined as follows: Stewardship was defined as the presence of health research resources and data availability to conduct BoD studies at a national level. Financing was defined as the presence of national agencies and funding to support national research. The availability of both human and physical resources to conduct research was considered, while the translation of research knowledge to inform health policy and strategies was considered as the fourth function.

Data for this study were gathered through the development of a set of 20 key questions by the lead researcher (SC) following literature review, with the aim of targeting the research landscape. These key questions were distributed among all management committee COST members (*n* = 12) representing the small states. Each COST member was responsible for completing the respective small country’s questionnaire themselves or by interviewing colleagues. Considering that most members form part of the public health research/academic/governmental bodies of the respective countries, such a task was easily conducted.

The 20 questions were categorized into three major themes to inform the operational components of each function, as follows:

(i) Stewardship: by asking seven questions related to the conduct of BoD studies at the national level, including any advantages or challenges faced by small states; for example: “*Has your country ever conducted a national/local BoD study? If yes, please state.*”
(ii) Data accessibility and ethical standards and available resources: by asking six questions related to research data accessibility including the availability of mortality and other morbidity registers and funding opportunities; for example: “*Does your country have a dedicated research hub/researchers employed to conduct national studies including health examination surveys, health interview surveys, BoD studies? Please provide details and specify.*”
(iii) Research translation and communication: by asking seven questions on the knowledge translation opportunities or challenges at the national level; for example: “*If a national/local BoD study was conducted, were the results (knowledge) translated into policy? Policy-makers used the results in actions/policies*?”

As part of this questionnaire, members were asked to provide examples of national surveys and studies conducted in recent years on communicable and noncommunicable diseases (NCDs). The answers were interpreted as a measure of the country’s capacity to conduct national research, as well as to establish the availability and accessibility of morbidity data for BoD studies. It also enabled us to identify the needs for disease-specific BoD studies in these small states. Each member was responsible for providing nationally representative information, attitudes and perspectives as well as associated literature, if any, related to these issues. All gathered data were then qualitatively analysed by comparing the small states on all key questions. The full questionnaire can be found as part of Additional file 1.

For the purposes of this study, knowledge translation was defined as the process of applying BoD knowledge generated or adopted within the local small-state context for the successful dissemination of such evidence to public health behaviour change, practice or policy [17].

## Results

### The research landscape in small states

Concerning stewardship, except for Latvia, the small states in Europe share a similar research landscape, with the health ministry or the national institutes of public health having a mandate to conduct national studies.

Regarding financial support and national priorities, not all states have a dedicated national budget, as performing BoD studies is not considered a national research priority (Fig. 1). Although some surveys are conducted on a regular basis, funding of these studies is usually through national funding agencies or third parties or universities. This is the case in Montenegro, where the Institute for Public Health depends primarily on a partner or a sponsoring institution for funding. Similar to other countries, Montenegro’s statistics office has an allocated budget for the conduct of regular surveys as well as population sampling and assisting the Institute for Public Health in other methodological aspects. Malta presents a similar scenario, where the Directorate for Health Information and Research, with the assistance of the National Statistics Office, carries out epidemiological studies, though most of the national studies are conducted by independent researchers at the University of Malta.

In terms of data availability and accessibility, various national mortality and cancer registers are available across almost all of the small states. All mortality registers follow the International Classification of Diseases, 10th revision (ICD-10), thus making the registry data suitable for use in BoD studies after quality control by national officers. The data from mortality registers can be used to calculate the YLL. In addition, small states maintain different disease-specific registers, which prevents them from performing comparative analyses of BoD assessment for a specific disease. Furthermore, some registries are not regularly updated or data collection has only been initiated in recent years. Regardless, data are readily available if the purpose of data use is appropriate and once all permissions (including ethical and data protection) have been obtained.

Given the small population size, data tend to be nationally representative, although data integrity is sometimes questionable, especially in those small countries that have difficulty in performing regular surveys and face problems in completing data collection. However, as is the case of Estonia, where the longitudinal monitoring of all medical consultations for individuals has been possible since 2002, extensive digitalization has enabled the automatization of the first step of data collection and aggregation for BoD analysis. This allowed for the inclusion of a range of parameters needed in the BoD calculations, which could then be supplemented with data from population surveys and other sources. Similarly, in Iceland, all healthcare encounters can be longitudinally followed up; however, these data have never been used to perform national BoD studies. Nonetheless, this demonstrates that Iceland is equipped in this regard to undertake its own national BoD studies.

Lack of expertise and resources (financial, human and infrastructure) are the main challenges faced by small states in conducting their own national BoD studies. However, it was noted that small states have several advantages. Indeed, the small geographical and population size gives them a head start if they choose to conduct national BoD studies. Considering these demographic features and the close-knit relationship between researchers and public health institutes, accessing data is easier, even if studies are conducted by independent researchers. Data collection, management and evaluation, if adequately resourced, are also easier for small states. Additionally, the presence of a centralized health system with linkable data sources may provide data representative of the entire population, as in the case of Estonia and Iceland. It appears that small states are in real need of national BoD studies covering NCDs, as these contribute to a substantial morbidity and mortality burden for each small state.

Regarding knowledge translation, public health officials and researchers in small states tend to have a close-knit relationship with the governing body and policy-makers. Such relationships can offer an advantage in knowledge translation, as noted by Estonia, Iceland, Luxembourg and Malta. Indeed, in Luxembourg, strategic planning incorporating research activities has already been implemented to follow the “bench-to-bedside” research concept. However, not all small states have embraced this concept. The small size of the country is sometimes a barrier because human resources are limited, with small teams responsible for various outputs, leaving little room for the practise of knowledge translation, as seen in Slovenia. Additionally, the lack of understanding of the concept of knowledge translation by some researchers, policy-makers and politicians, as well as the lack of tools to measure knowledge translation, hinders the actual knowledge translation process. Another challenge faced by researchers in translating research knowledge to aid policy-makers and politicians occurs when the results are not aligned with the political agenda or interests of the respective parties. It is especially relevant in the case where the research findings recommend reorganization of systems or allocation of funds to sections that are not part of the policy/political agenda.

Nevertheless, among the challenges countries face in knowledge translation, some successful stories were noted. In Estonia, the results of the first national BoD study were used to shape the National Health Plan 2009–2020, with members of the national BoD team being part of the core team tasked with developing the national policy. Another positive knowledge translation experience is the implementation of the Icelandic Prevention Model. This was based on results of a national study that reduced adolescent drug use in Iceland and secured significant funding from the European Research Council in 2015 to further explore adolescent health and behaviour and to develop a proof of concept in primary prevention in 2022. Results from the first national COVID-19 BoD study in Malta contributed to securing funding from the European Union (EU) for further COVID-19 testing.

## Discussion

Stellar healthcare systems with the provision of optimal healthcare are dependent on effective and up-to-date knowledge-to-action frameworks. This relies on the translation of research findings into practice, also known as knowledge translation [17, 18]. Therefore, regular health and disease-specific national research studies must be conducted to ensure the availability of evidence-based data. BoD studies are an excellent source of research to provide such evidence; however, the evidence–practice policy gap is still evident, as this study indicates.

Our analysis revealed that health research systems in these small countries are fragmented, competitive, highly topic-specialized and reliant on sectoral activities. For example, biomedical researchers, clinicians, epidemiologists, health systems researchers, health statisticians, social and behavioural scientists, and health economists often work in isolation. There is insufficient communication between the producers of research findings and decision-makers and the beneficiaries.

Small states are generally overlooked in the global research agenda in discussions of knowledge translation and in obtaining resources for conducting BoD studies. Indeed, the high prevalence of NCDs in these small countries is evidence of the need for BoD studies. For example, in Luxembourg, a high prevalence of cardiometabolic diseases has been well documented during the last decade [19], but addressing the burden of cardiometabolic disease is not considered a public health priority for funding agencies. This is also the case for Cyprus, where 90% of all mortality is attributed to NCDs, and there is an imbalance between research funding and output [20]. Therefore, conducting national BoD studies should highlight the true burden of these NCDs and allow for the reformulation of public health priorities. Similarly, in Latvia, cancer treatment is only partially covered by the government, and HIV is a widespread communicable disease [21]. Another communicable disease is hepatitis C, which is highly prevalent among drug abusers in Montenegro [22]. Hence, BoD studies covering these diseases would elucidate the burden of these diseases at a population level and provide evidence for the action needed in relevant areas. There is currently no BoD study planned for any of these small states.

As noted above, with the right tools and resources, it is possible to conduct national BoD studies in small states [14]. Indeed, some of the small countries have conducted selected national BoD studies. For example, Malta conducted a national BoD study for low back pain and estimated the direct effects of COVID-19 on DALYs during the first year of the pandemic, with the help of the recently established European Burden of Disease Network [23, 24], while Montenegro is part of the BoCO-19 project (Burden of Disease due to COVID-19—Towards a harmonization of population health metrics for the surveillance of dynamic outbreaks; project number D81905), which aims to calculate BoD indicators for COVID-19 [25]. Nevertheless, only Estonia has conducted a national and subnational BoD estimate exercise [26], and since 2013 these have been carried out biannually, with results publicly available in the national database of health statistics and survey results [18]. However, limited human resources and lack of funding are a challenge unless funds can be obtained from third parties. Therefore, up-to-date evidence generated or adopted by policy-makers and politicians is needed for use in practice and policy. Indeed, a common theme was identified among small states where the generation or transfer of knowledge through the conduct of research is perceived as the real “bottleneck” in the knowledge translation process.

Small states are seen as having a unique advantage in translating knowledge into practice and policy more easily, given their size and knowledge transfer between them. The direct results of knowledge translation can also be more easily and quickly discernible. However, several challenges remain. One hindrance is the absence of or limited knowledge translation culture in some small states. Therefore, improving the understanding of knowledge translation at the national level through training sessions is encouraged. Additionally, the creation of model knowledge translation frameworks specific to small states could help overcome these barriers and create channels of communication between researchers and policy-makers or decision-makers. Therefore, identifying the best mode of knowledge translation, such as the use of infographics [27], could enable stakeholders and policy-makers to understand and use the research findings. Further, identifying the specific challenges and highlighting the importance of knowledge translation in small states should facilitate the knowledge translation process.

This descriptive study has both strengths and limitations. To our knowledge, this is the first attempt to shed light on the research landscape, including BoD studies and knowledge translation processes, in small states. The study is dependent on personal communication efforts between the members of the small states involved and the relevant institutions in the respective countries that oversee data collection and registries, which may be considered a limiting factor. More sustainable efforts should be made to reinforce the communication between researchers and key stakeholders by creating processes that capture all aspects of data use and knowledge generation and adoption at a higher level of the knowledge translation processes. However, the associated strength is that this study has provided a direct understanding of the organizations involved and their capacity and culture with respect to the implementation of national BoD studies. This study can therefore contribute to the mapping of processes around the stakeholders involved in each small state and the development of specific protocols tailored to the conduct of BoD, using available local resources and further enhancing the knowledge translation culture.

### Implications for practice and recommendations

Traditionally, capacity development interventions have been overly reliant on big-ticket events such as face-to-face training and workshops. Based on our deep understanding of the problems of small countries with respect to BoD studies, the suggested capacity development interventions can range from expert-driven consultancy services, to virtual or face-to-face training and peer-to-peer exchanges. Cross-country sharing of experiences can foster beneficial synergistic partnerships in health research. Additionally, a more active collaborative role should be taken by small-country collaborators in the IHME GBD studies by providing national data, learning GBD methods, reviewing national estimates and using the GBD estimates for knowledge translation, while at the same time developing national BoD studies for diseases and risks and comparing their results with the GBD results until an independent BoD approach is sufficiently developed.

## Conclusion

Small states, if equipped with adequate human, infrastructural and financial resources, may have the capacity to conduct national BoD studies. The participation of all stakeholders primarily involved in knowledge generation and translation in the public and private sectors is a key to success. Indeed, the findings of this work can serve as a model for identifying current gaps and opportunities for each of the eight small European countries as well as a guide for performing country BoD studies. In addition, it should be acknowledged that with proper implementation, the small states could serve as an ideal setting for pilot BoD studies and as a model for translation of results into health policy. Such an implementation study can provide a valuable contribution to BoD health research by providing benchmarks and information on best practices and lessons learned, and can potentially be beneficial to larger regions or countries. Ultimately, this study can serve as a road map for translating country-specific knowledge about BoD work through local stakeholder engagement, health research prioritization and health policy development.

In sum, this article provides an overview of the unique characteristics and context of small states, with the goal of assisting national and international authorities in working towards successful national BoD studies and knowledge translation. This can benefit the local population and ultimately contribute to the use of data to guide practice and policy.

## Supplementary Information

**Additional file 1:** The 20-questions questionnaire used to collect data among the authors.

## Data Availability

The datasets used and/or analysed during the current study are available from the corresponding author on reasonable request.

## Abbreviations

BoD: Burden of disease
DALY: Disability-adjusted life-years
GBD: Global Burden of Disease study
NCDs: Noncommunicable diseases
YLD: Years lived with disability
YLL: Years of life lost
