# Supplementary material for: Conducting national burden of disease studies and knowledge translation in eight small European states: challenges and opportunities
Source: Health Res Policy Syst. 2022 Oct 21;20:113. doi: 10.1186/s12961-022-00923-1 (PMC9587663; doi:10.1186/s12961-022-00923-1)
Supplement: Supplementary file 1 — Additional file 1: The 20-questions questionnaire used to collect data among the authors. [file 12961_2022_923_MOESM1_ESM.docx]

| **Sections** |  | **Questions** |
| --- | --- | --- |
|  |  |  |
| Stewardship  [Burden of Disease (BoD) Studies] | 1 | Are there experts in conducting BoD studies in your country? |
|  | 2 | Has your country ever conducted a national/local BoD studies? If Yes, please state what the BoD study was about and provide references |
|  | 4 | Has a BoD study been conducted or is being conducted to assess the burden of COVID-19? If yes, please provide details |
|  | 5 | What advantages do small states have in conducting BoD studies? Please specify |
|  | 6 | What challenges do you think small states face in conducting BoD studies? Please specify |
|  | 7 | Which BoD Studies would your country benefit from? (For example on NCD/communicable disease/injury) Please specify the reasoning behind this as well as whether such study has already been conducted or is in the pipe-line or there are no plans for. |
| Data accessibility, ethical standards and available research | 1 | Does your country have a dedicated research hub / researchers employed to conduct national studies including health examination surveys, health interview surveys, BoD Studies? Please provide details and specify. Any references to past studies would be appreciated. |
|  | 2 | In your country, who has the mandate to conduct national studies? |
|  | 3 | Is a mortality register available in your country? Does it use the ICD10 classification? |
|  | 4 | Are registers for NCDs, communicable diseases and/or Injuries present in your country? Please specify |
|  | 5 | Is access to data/results of population based surveys or access to hospital records permissible? (In order to conduct BoD studies) Please specify which databases are available and provide details about accessibility, quality of data, national representative? |
|  | 6 | Is there a dedicated national budget allocated to conduct BoD studies / any other national research? |
| Research translation and communication | 1 | What challenges are faced by small states in Knowledge translation (KT) especially to translate research results to policy-makers & politicians? Please specify and give examples if applicable |
|  | 2 | How open are policymakers to take up new research results / BoD estimates? |
|  | 3 | If a national/local BoD study was conducted, were the results (knoweldge) translated into a policy? Policy makers used the results into actions/policies? |
|  | 4 | Do you have any recommendation/s on how KT can be improved in your country? If you have any examples including any local reports, please provide link or reference |
|  | 5 | In the last 2 years, since the onset of COVID-19, has your country or any researcher/s from your country has/have conducted a national study where the results were translated into policies? I.e. evidence of successful KT. If so, can you elaborate & provide reference/s |
|  | 6 | Do you think that KT should be highlighted more especially within small states. Please provide reasons & references where appropriate |
|  | 7 | Does your country use a KT framework adopted from a larger country? If so, can you specify which country and any reasons pertaining to such practice |
